# Changes in Extremely Hot Summers over the Global Land Area under Various Warming Targets

**DOI:** 10.1371/journal.pone.0130660

**Published:** 2015-06-19

**Authors:** Lei Wang, Jianbin Huang, Yong Luo, Yao Yao, Zongci Zhao

**Affiliations:** Ministry of Education Key Laboratory for Earth System Modeling, Center for Earth System Science, and Joint Center for Global Change Studies (JCGCS), Tsinghua University, Beijing, China; University of California San Diego, UNITED STATES

## Abstract

Summer temperature extremes over the global land area were investigated by comparing 26 models of the fifth phase of the Coupled Model Intercomparison Project (CMIP5) with observations from the Goddard Institute for Space Studies (GISS) and the Climate Research Unit (CRU). Monthly data of the observations and models were averaged for each season, and statistics were calculated for individual models before averaging them to obtain ensemble means. The summers with temperature anomalies (relative to 1951–1980) exceeding 3σ (σ is based on the local internal variability) are defined as “extremely hot”. The models well reproduced the statistical characteristics evolution, and partly captured the spatial distributions of historical summer temperature extremes. If the global mean temperature increases 2°C relative to the pre-industrial level, “extremely hot” summers are projected to occur over nearly 40% of the land area (multi-model ensemble mean projection). Summers that exceed 5σ warming are projected to occur over approximately 10% of the global land area, which were rarely observed during the reference period. Scenarios reaching warming levels of 3°C to 5°C were also analyzed. After exceeding the 5°C warming target, “extremely hot” summers are projected to occur throughout the entire global land area, and summers that exceed 5σ warming would become common over 70% of the land area. In addition, the areas affected by “extremely hot” summers are expected to rapidly expand by more than 25%/°C as the global mean temperature increases by up to 3°C before slowing to less than 16%/°C as the temperature continues to increase by more than 3°C. The area that experiences summers with warming of 5σ or more above the warming target of 2°C is likely to maintain rapid expansion of greater than 17%/°C. To reduce the impacts and damage from severely hot summers, the global mean temperature increase should remain low.

## Introduction

With rapid increases in global mean temperature over the past decade, climate extremes have become stronger and more frequent [1–4]. Particularly, heat waves, which severely threaten human health and property, have garnered much attention [5]. For example, the European heat waves in 2003 and 2006 [6–8], the great California heat wave in 2006 [9], the rare high temperatures in Russia in 2010 [10, 11] and the extremely hot Australian summer in 2013, are all well-known for their severity and damage. Additionally, studies have indicated that heat waves will occur with higher intensity and frequency if global warming continues [12–17].

Heat waves also occur on monthly and seasonal scales as temperature extremes [18–22]. Because monthly data are relatively easy to access, monthly temperatures are widely used to examine changes in past and future climate extremes [23]. The number of warm monthly temperature extremes has significantly increased in the past decade [24, 25]. Hansen et al. [18] indicated that the occurrence of extremely hot summers, which rarely occurred in 1951–1980 (less than 1% of the land area), became extensive in the last decade (approximately 10% of the land area). In addition, the latest results from the fifth phase of the Coupled Model Intercomparison Project (CMIP5) models suggest that extremely hot monthly temperatures will affect approximately 85% of the land area in 2100 under the Representative Concentration Pathway 8.5 (RCP8.5) [19].

A global mean temperature increase of 2°C relative to pre-industrial level has been accepted by most countries and organizations as a critical target because it seems achievable and would limit climate and ecological impacts [26–30]. Controlling the increase in the global mean temperature is as an effective method for avoiding or reducing losses caused by global warming. Consequently, quantifying the responses of climate extremes to a 2°C warming and other warming rates relative to the pre-industrial level will help policymakers, researchers and the public understand the possible consequences of inaction. The seasonal temperatures of the 26 CMIP5 models, which include historical simulations and three RCP scenarios, were analyzed in this study. Compared with the ocean, land areas are more closely associated with human livelihood. Accordingly, we focused on changes in temperature extremes over global land areas in response to various warming targets. Summer is the focus of our research because humans are more sensitive to hot extremes during the summer than during other seasons.

Hansen et al. [18] and Coumou and Robinson [19] also research on the extreme temperatures. However, Hansen et al. [18] only investigated the observations by using GISS data, while Coumou and Robinson [19] mainly predicted the area percentage with monthly heat extremes during boreal summer in 21st century and their spatial patterns at the end 20 years of 21st century under RCP2.6 and RCP8.5 scenarios. In contrast, based on 26 CMIP5 models, a systemic evaluation of the long-term global extreme summer temperatures was performed to quantify the capability of models in simulating extreme summer temperatures in our study, including not only the spatial distributions and total area percentages but also the probability of occurrence. The global extreme summer temperatures include June-July-August (JJA) in the Northern Hemisphere and December-January-February (DJF) in the Southern Hemisphere, while boreal summer of JJA was analyzed in both hemispheres in Coumou and Robinson [19]. In addition, we mainly focus on the response of extremely hot summers to the different warming magnitudes relative to the pre-industrial level. Therefore, all simulations from RCP2.6, RCP4.5 and RCP8.5 scenarios (actually 26 simulations in each RCP scenario) are together used in our research to quantify the various warming magnitudes regardless of different emission scenarios. So our analysis emphasizes the response of summer climate extremes to different warming targets rather than different emission scenarios as Coumou and Robinson [19]. Furthermore, another big difference between this work and Coumou and Robinson [19] is that the seasonal data are used in this study instead of monthly data in the research of Coumou and Robinson [19]. Since the standard deviation of monthly data is larger than that of seasonal data, the projected future increase of the hot extreme temperatures analyzed by using seasonal data is generally larger than those analyzed by using monthly data.

This paper is structured as follows. Section 1 introduces the data and methods used in this research. Next, 26 CMIP5 models are evaluated for simulating historical temperature extremes in section 2. In section 3, the responses of the temperature extremes to various warming targets are analyzed. Finally, a summary and discussion of our finding is presented in section 4.

## Data and Methods

Monthly surface air temperatures from 26 CMIP5 models, which include historical data and three simulated scenarios (RCP2.6, RCP4.5 and RCP8.5) (S1 Table), were used in our research. Historical observations were obtained from the Goddard Institute for Space Studies (GISS) [31] and the Climate Research Unit (CRU) [32]. The monthly GISS data (1880 to the present), which are smoothed to 250 km to include as much regional variability as possible, have a spatial resolution of 2°x 2° [31], and the monthly CRU data (1901 to the present) have a high spatial resolution of 0.5° x 0.5° [32]. The durations of the 26 CMIP5 historical runs are shown in S1 Table, most of which extend from 1850 to 2005. Most of the 21^st^ century projections, which follow the three emission scenarios, extend from 2006 to 2100.

Monthly data are averaged for each season at the beginning, and the summer means of JJA data from the North Hemisphere and DJF data from the South Hemisphere are concurrently analyzed in the following.

In our research, the period of 1951–1980 was chosen as a reference period because of its advantages. As mentioned by Hansen et al. [18], the temperature during this period was relatively stable and almost without rapid increase, which quite a number of older people have experienced.

The definition of temperature extremes used in this study is similar to that proposed by Hansen et al. [18], except that a value of +5σ is included in our research. In a normal distribution, the values between ±0.43σ are defined as “typical” summers; the values greater (less) than +0.43σ (-0.43σ) are defined as “hot” (“cold”) summers; the values greater (less) than +2σ (-2σ) and +3σ (-3σ) are defined as “very hot” (“very cold”) and “extremely hot” (“extremely cold”) summers, respectively. As global warming continues, “extremely hot” events will become more common. Additionally, changes in the occurrence of more severe summers have been quantified: the summers with temperature anomalies greater than +5σ in a normal distribution are referred to as “exceeding 5σ hot” summers.

To calculate the local σ, we first fit the summer data from 1901 (the first year of CRU data) to 2005 using a least squares polynomial method and determined the long-term non-linear trend. Next, the summer time series were detrended by deducting the long-term trend. Then, we estimated σ by using this detrended 105 data, which include a sufficient number of data points to estimate the local year-to-year variability.

Considering the starting time of the historical simulations of the 26 models (S1 Table), the average temperature from 1861 to 1880 is used to represent the pre-industrial level. When the 11-year running mean global mean temperature exceeds 2°C, the global mean temperature reaches the 2°C target. Additionally, the 20-year climate centered on the year reaching 2°C target is determined. The same methods are used to examine the climate when the global mean temperature reaches various warming targets relative to the pre-industrial level, such as 3°C, 4°C and 5°C. In this analysis, the statistical characteristics of the ensemble mean of the 26 models are obtained after calculating each model independently by considering the weight of each grid area. That is to say, the statistics were first calculated for individual models, and then the results from all models were averaged to present the multi-model ensemble mean. Additionally, JJA in the Northern Hemisphere and DJF in the Southern Hemisphere are considered jointly when presenting the summer characteristics.

## Results

### Evolution of historical summer temperature extremes

**Fig 1:**
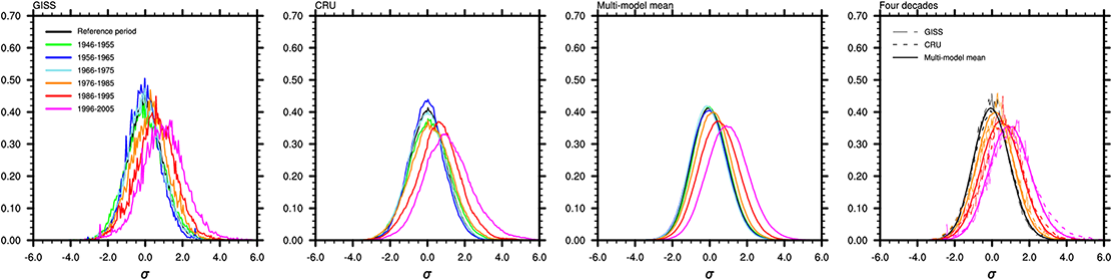
Frequency of summer local temperature anomalies. The temperature anomalies (relative to the 1951–1980 mean) are divided by the local standard deviation of 1901–2005 and binned into intervals of 0.05. The local standard deviation is calculated from the long-term non-linearly detrended time series (details in data and method section). For the multi-model ensemble mean, this process was conducted for each model, and the final frequencies were averaged. The first, second and third graphs show the GISS, CRU and multi-model ensemble mean results, respectively. To better compare the results from observations and multi-model ensemble mean, the frequencies during the periods of 1951–1980 (reference period), 1976–1985, 1986–1995 and 1996–2005 are shown in the fourth graph.

Comparing the results from the multi-model ensemble mean and two observations of GISS and CRU reveals that the CMIP5 models perform extremely well when reproducing the evolution of the statistical distribution of summer temperature extremes between 1946 and 2005 (Fig 1). Because the historical CMIP5 runs end in 2005, the temperature extremes are counted every ten years from 1946 to 2005 for the model results and two observational datasets. The curves of the summer temperatures are smoother in the CRU dataset than in the GISS dataset because of the additional observational grids in the CRU data. The distributions of the multi-model ensemble mean are highly consistent with those of the observations. The curves shift to the right and become broader over time, particularly following the 1980s. The probability of an “extremely hot” summer is indicated by a gradually increasing area of the right tail. The curve shifts rightward as the mean temperature increases, and the broader trend may result from the heterogeneous warming rates in different regions rather than the increase of variability [33]. Fei Ji et al. [34] indicated that the most dramatic and rapid warming has occurred in arid or semiarid regions in the Northern Hemisphere at mid-latitudes, and that warming since 1900 has varied spatially and unevenly. However, the global temperature variability has remained stable [35]. As shown in Fig 1, rapid warming has occurred since the 1970s, which resulted in significant rightward curve shifts. In addition, the spatial combined statistics from regions with different warming rates contributes to the broadening of the curves. The occurrence probability of an “extremely hot” summer during each period is shown in Table 1, and the occurrence probability is somewhat underestimated by the multi-model ensemble mean.

**Table 1:** Occurrence probability of an “extremely hot” summer over the global land area.

Period	GISS (%)	CRU (%)	Multi-model ensemble mean
Reference period	0.1	0.1	0.1
1946–1955	0.3	0.5	0.3
1956–1965	0.1	0.1	0.1
1966–1975	0.1	0.2	0.2
1976–1985	0.6	0.7	0.4
1986–1995	1.7	2.0	1.4
1996–2005	4.8	5.1	4.3

The second, third and fourth columns in the table present results from the two observational datasets and multi-model ensemble mean.

**Fig 2:**
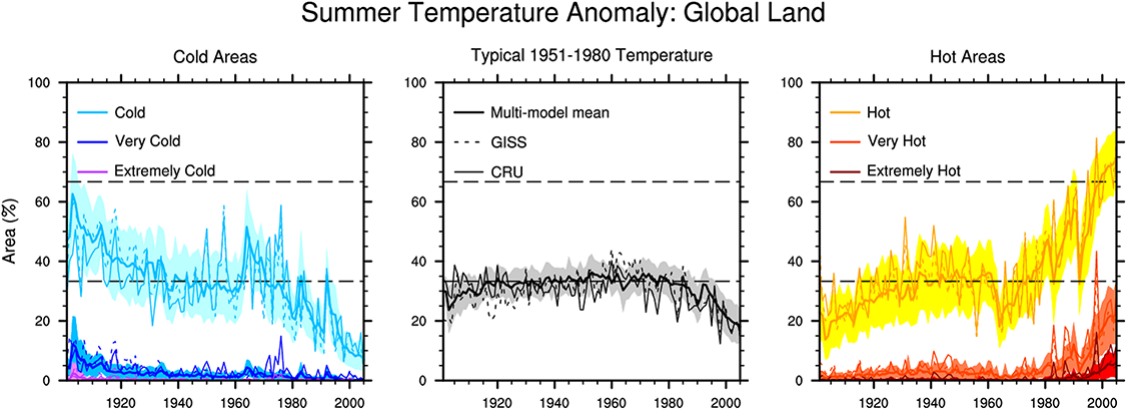
Percentages of global land areas hit by summer temperature extremes. The time series spans from 1901 to 2005. The thick lines are the results of 26 individual models averages. The shading in the plots denotes the 1σ uncertainty range of the 26 CMIP5 models.

**Fig 3:**
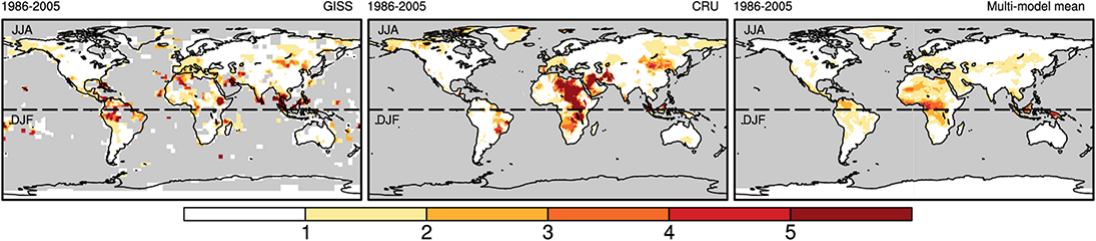
Spatial distribution of the “extremely hot” summers frequency from 1986 to 2005. The left, middle and right maps represent the GISS, CRU and averages of the individual models results, respectively.

The multi-model ensemble mean performs well in terms of the total area affected by temperature extremes (Fig 2). The “extremely hot” area has rapidly increased since the late 1970s, and the “cold” area has generally decreased, with the exception of a relatively stable period from the early 1940s to the mid-1970s. The trends in the hot and cold areas are highly consistent with those of the observations. The averaging process results in smoother multi-model ensemble mean curves than observed for the other two observations. The spatial distributions of the “extremely hot” summers during 1986–2005 are shown in Fig 3. When disregarding the missing data in GISS, the locations with “extremely hot” summers are similar between the two observations, except for the west of South America and the middle of Africa. The present spatial distribution and areal coverage of “extremely hot” summer that are simulated using the multi-model ensemble mean is more similar to the GISS results than the CRU results, in which important locations, such as West Europe, South America and North Africa, are all significant, but their exact frequency of “extremely hot” summers is underestimated.

### Response of hot summer extremes to various warming targets

The multi-model ensemble mean well reproduces the statistical characteristics of the probability density distribution and covering area percentage of the present temperature extremes over the global land area, and partially captures the locations of “extremely hot” summers. This systematic evaluation of the performance of the models on hot summer extremes increases the confidence in the projected future changes in temperature extremes. Under the RCP2.6 scenario, the global mean temperatures of 8 models (8/26) reach the 2°C target, even though this scenario is designed to limit the global mean temperature increase to within 2°C relative to the pre-industrial level. A 3°C warming target was reached by 8 models in the RCP4.5 scenario and by all 26 models in the RCP8.5 scenario. Regarding the 4°C and 5°C warming targets, 19 and 9 models, respectively, from the RCP8.5 scenario reached these targets. In this research, we only focused on the response of the temperature extremes to different magnitudes of warming relative to the pre-industrial level. Thus, the emission scenarios (RCP2.6, RCP4.5 and RCP8.5) are not distinguished in the analysis. As a result, 78 simulations are used, from 26 CMIP5 models, each simulating the three scenarios.

**Fig 4:**
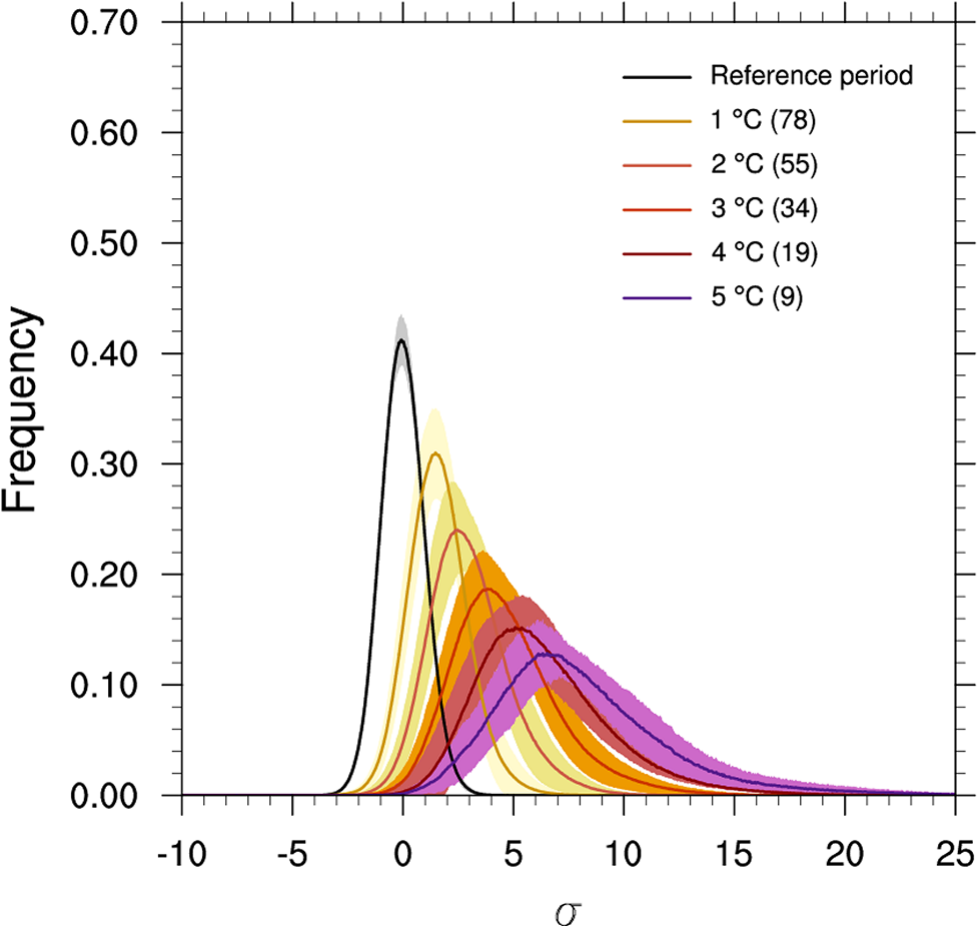
Frequency of local summer temperature anomalies. These results are based on different magnitudes of warming relative to the pre-industrial level. The temperature anomalies (relative to 1951–1980) are divided by the local standard deviation of 1901–2005 and binned into intervals of 0.05 for individual models before averaging the statistical results. The local standard deviation is calculated from the long-term non-linearly detrended time series (details in data and method section). The total number of simulations that reach the warming targets is shown in brackets. The shading represents the 1σ range of uncertainty.

As shown in Fig 4, the curves of the probability density widen and shift right as the global mean temperature increases. Thus, “extremely hot” summers will occur more readily in the future. The rightward shift is more severe than that during the 1990s due to the gradual aggravation of global warming, and significant broadening may result from various warming rates in different regions [33, 35]. The details regarding changes in probability are shown in Table 2. Notably the occurrence probability of an “extremely hot” summer would increase to 40.3% as the global temperature reaches the 2°C warming target and 93.3% as the global temperature reaches the 5°C warming target. The probability was only 0.1% during the reference period. When the increase of the global mean temperature exceeds 2°C or 5°C relative to the pre-industrial level, the occurrence probabilities of having an “exceeding 5σ hot” summer may increase to 12.9% and 79.6%, respectively. This probability was zero during the reference period.

**Table 2:** Probability of “extremely hot” (>3σ) and “exceeding 5σ hot” (>5σ) summers in response to different magnitudes of warming relative to the pre-industrial level.

Warming targets	>3σ (%)	>5σ (%)
Reference period	0.1	0
1°C	11.0	1.3
2°C	40.3	12.9
3°C	70.4	39.1
4°C	87.3	64.0
5°C	93.3	79.6

**Fig 5:**
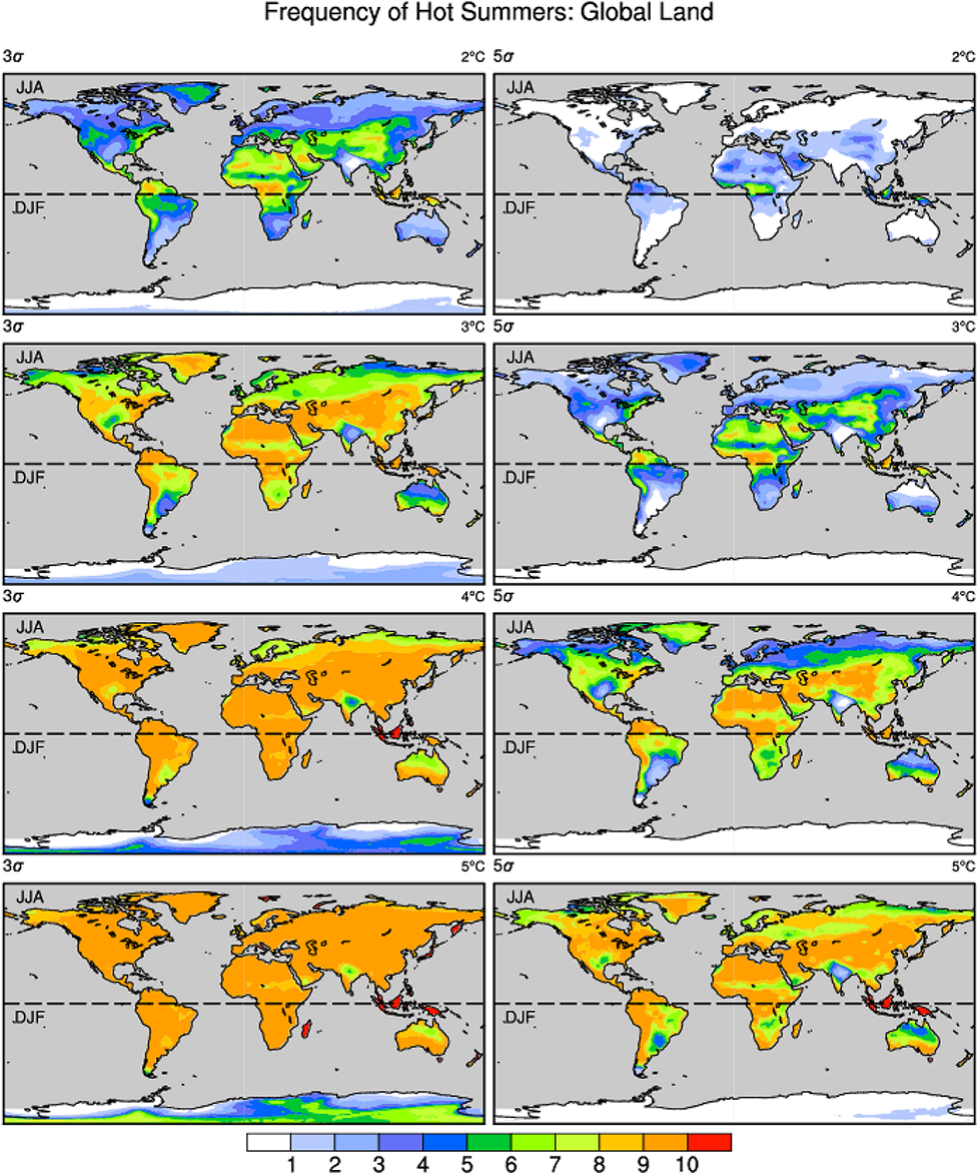
Spatial distribution of the frequency of “extremely hot” (>3σ) and “exceeding 5σ hot” summers. The maps show the frequency of “extremely hot” and “exceeding 5σ hot” when the temperature increases by from 2°C to 5°C relative to pre-industrial level. The σ is calculated from the long-term non-linearly detrended time series (details in data and method section). The left and right columns present the “extremely hot” and “exceeding 5σ hot” summers at different warming targets (2°C, 3°C, 4°C and 5°C in rows 1, 2, 3, and 4, respectively). The upper and lower halves of each plot describe the boreal summer (JJA) and austral summer (DJF), respectively. The color bar is different from that shown in Fig 3. Units: times/10 yrs.

Which regions will be vulnerable to hot summer extremes in the future when warming targets are reached? The frequencies of “extremely hot” and “exceeding 5σ hot” summers in response to different warming rates (2°C to 5°C) relative to the pre-industrial level are shown in Fig 5. When the 2°C target is reached, an “extremely hot” summer is projected to occur twice every ten years at most low-mid latitudes. Central and western Asia, central and northern Africa and northern South America are relatively more vulnerable than other areas. Furthermore, many areas seem to experience “extremely hot” summers 7 out of 10 years and may even suffer an “exceeding 5σ hot” summer. When the 3°C warming target is reached, this situation becomes worse. Both “extremely hot” and “exceeding 5σ hot” summers are projected to occur more frequently at a 3°C than at a 2°C warming. Nearly all land areas would experience “exceeding 5σ hot” summers during a 10-year period, excluding Australia. When the global mean temperature reaches the 4°C and 5°C warming targets, essentially no land areas would be excluded from “extremely hot” summer every year. Warming by 5°C relative to pre-industrial level would also make “exceeding 5σ hot” summers extremely common in most land areas. Notably, the impacts on Australia, India and Antarctica are relatively small compared with other locations.

**Fig 6:**
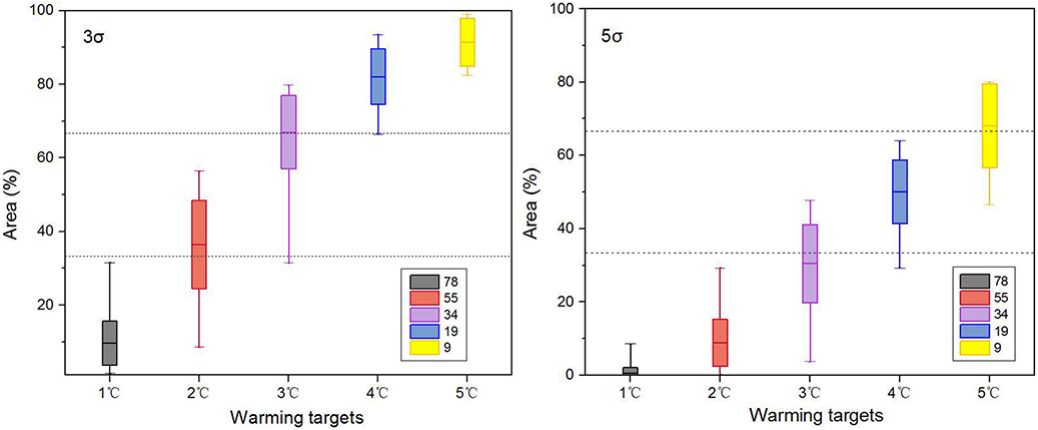
Percentages of the areas with “extremely hot” (left) and “exceeding 5σ hot” (right) summers. The maps show the responses to different magnitudes of warming relative to pre-industrial level. The σ is calculated from the long-term non-linearly detrended time series (details in data and method section). The colors indicate the number of simulations. The line in the middle of each box represents the ensemble mean of the individual model results, and the upper and bottom edges of the box show the range of the 1σ uncertainty. The two bars outside the box represent the maximum and minimum simulation values.

The responses of the areas affected by “extremely hot” and “exceeding 5σ hot” summers to different warming targets are specifically examined based on 78 CMIP5 simulations (Fig 6). All 78 simulations reached the 1°C warming target, and 55, 34, 19 and 9 of the simulations reached the 2°C, 3°C, 4°C, 5°C warming targets, respectively. When the 1°C warming target is reached, 9.7% of the global land is projected to experience an “extremely hot” summer, and an “exceeding 5σ hot” summer would rarely occur over land. After exceeding the 3°C warming target, more than half of the global land area (67.0%) could experience an “extremely hot” summer. In addition, almost all of the land areas (91.4%) would experience “extremely hot” summers when the 5°C warming target is reached. “Exceeding 5σ hot” summers are projected to occur over more than half of the land area when the 4°C warming target is exceeded. Notably, the area affected by “extremely hot” summers expands rapidly between warming targets of a 1°C and 3°C, with an area increase of more than 25% per degree. Above the 3°C warming target, the area increases by less than 16% per degree. However, the areas that experience “exceeding 5σ hot” summers continues to rapidly increase by more than 17% per degree after the 2°C warming target is reached.

## Summary and Discussions

Global warming may result in various impacts on the ecology and environment. Our research focused on the changes in summer temperature extremes over land areas around the world in response to different magnitude of warming, showing one aspect of impacts from global warming. To use the models’ future prediction for quantifying the temperature extremes, their simulating ability need to be evaluated first. A systematic evaluation of the performance of the models on predicting hot summer extremes showed the multi-model ensemble mean of the 26 CMIP5 models well reproduced the statistical characteristics of historical summer temperatures and captured the main locations of “extremely hot” summers. The performance of the multi-model ensemble mean is important for projecting future changes in extreme summer temperatures. The frequency, areal extent and intensity of summer temperature extremes are all projected to increase as global warming continues and higher warming results in more severe hot summers. Mid-low latitude areas are projected to experience more summer temperature extremes than high latitudes. Exploring the response of summer temperature extremes to various warming targets will improve our understanding in the importance of controlling the magnitude of warming. To reduce the impacts and damage from severely hot summers, global warming should be limited as much as possible. The observations indicate that the global mean temperature has currently increased by 0.8°C since the industrial revolution [36]. Thus, stricter greenhouse gas emission policies should be enacted to mitigate global warming as soon as possible.

The quantification of temperature extremes relative to local climate mean and variability offers of useful way to analyze the extreme temperatures. Furthermore, distinct from heat waves defined by daily temperatures, this kind of definition emphasizes the overall climate change. The heat waves are of concern mainly because of their significant impacts on the human health, while hot extremes on seasonal scales may have a long-term impact on the ecological aspects, such as crop growth and animal breeding. However, one limitation of this definition method is to neglect the absolute temperature. For example, the standard deviations for most high latitudes in the Northern Hemisphere are relatively greater than those in the tropics, and the mean temperature is lower at the high latitude. When the 2°C target is reached, the temperatures exceeding 3σ in most of the high latitude areas remain less than 30°C, while the temperatures less than 3σ in some areas of tropics are higher than 30°C. Thus, this definition of temperature extremes cannot reflect the local absolute temperatures.

In addition, only the changes in the extremely hot summer temperatures are analyzed in this study, while the reasons for changes are not discussed. Some researchers have indicated that temperature extremes may be linked to weakening summer circulations, anomalous patterns of mid-latitudinal planetary waves, soil and atmospheric moisture, the interactions with precipitation extremes and other factors [7, 37–41]. The temperatures in different regions are affected by various circulation systems and other local characteristics. The mechanisms that cause the changes in extremely hot summers under various warming targets at different locations should be studied in the future.

## Supporting Information

S1 Table Affiliations and atmospheric model resolutions of the 26 CMIP5 models. The durations of the historical and RCP runs are also provided.
(DOCX)
