# Supplementary material for: Changes in Extremely Hot Summers over the Global Land Area under Various Warming Targets
Source: PLoS One. 2015 Jun 19;10(6):e0130660. doi: 10.1371/journal.pone.0130660 (PMC4474600; doi:10.1371/journal.pone.0130660)
Supplement: S1 Table — The durations of the historical and RCP runs are also provided. (DOCX) [file pone.0130660.s001.docx]

**Table 1. Affiliations and atmospheric model resolutions of the 26 CMIP5 models.**

| Sponsor, Country | CMIP5 models | Historical duration | RCPs duration |
| --- | --- | --- | --- |
| Beijing Climate Center (BCC), China | BCC-CSM1.1 (128×64) | 1850-2012 | 2006-2099 |
| Beijing Normal University (BNU), China | BNU-ESM (128×64) | 1850-2005 | 2006-2100 |
| Canadian Centre for Climate Modelling and Analysis (CCCMA), Canada | CanESM2 (128×64) | 1850-2005 | 2006-2100 |
| Centre National de Recherches Météorologiques (CNRM), France | CNRM-CM5 (256×128) | 1850-2005 | 2006-2100 |
| Norwegian Climate Center (NCC), Norway | NorESM1-M (144×96) | 1850-2005 | 2006-2100 |
|  | NorESM1-ME (144×96) | 1850-2005 | 2006-2100 |
| National Center for Atmospheric Research (NCAR), United States | CCSM4 (288×192) | 1850-2005 | 2006-2100 |
|  | CESM1-CAM5 (288×192) | 1850-2005 | 2006-2100 |
| Commonwealth Scientific and Industrial Research Organization (CSIRO), Australia | CSIRO-Mk3.6.0 (192×96) | 1850-2005 | 2006-2100 |
| Max Planck Institute (MPI), Germany | MPI-ESM-LR (192×96) | 1850-2005 | 2006-2100 |
|  | MPI-ESM-MR (192×96) | 1850-2005 | 2006-2100 |
| Institute of Atmospheric Physics (IAP), China | FGOALS-g2 (128×60) | 1850-2005 | 2006-2100 |
|  | FGOALS-s2 (128×108) | 1850-2005 | 2006-2100 |
| Geophysical Fluid Dynamics Laboratory (GFDL), United States | GFDL-CM3 (144×90) | 1860-2005 | 2006-2100 |
|  | GFDL-ESM2G (144×90) | 1861-2005 | 2006-2100 |
|  | GFDL-ESM2M (144×90) | 1861-2005 | 2006-2100 |
| Goddard Institute for Space Studies (GISS), United States | GISS-E2-R (144×90) | 1850-2005 | 2006-2100 |
| L’Institut Pierre-Simon Laplace (IPSL), France | IPSL-CM5A-LR (96×96) | 1850-2005 | 2006-2100 |
|  | IPSL-CM5A-MR (144×143) | 1850-2005 | 2006-2100 |
| First Institute of Oceanography (FIO), China | FIO-ESM (128×64) | 1850-2005 | 2006-2100 |
| Model for Interdisciplinary Research on Climate (MIROC), Japan | MIROC-ESM (128×64) | 1850-2005 | 2006-2100 |
|  | MIROC-ESM-CHEM (128×64) | 1850-2005 | 2006-2100 |
|  | MIROC5 (256×128) | 1850-2012 | 2006-2100 |
| Meteorological Research Institute (MRI), Japan | MRI-CGCM3 (320×160) | 1850-2005 | 2006-2100 |
| Met Office (UKMO), UK | HadGEM2-ES (192×145) | 185912-200511 | 2006-2100 |
|  | HadGEM2-AO (192×145) | 1860-2005 | 2006-2099 |

The durations of the historical and RCP runs are also provided.
